# The Moderating Role of Depression in the Relationship Between Religious Discrimination and Interpersonal Competence Among Turkish Psychiatric Immigrants in Germany

**DOI:** 10.1192/j.eurpsy.2026.11455

**Published:** 2026-07-31

**Authors:** E. D. Cindik-Herbrueggen, E. N. Tuyluoglu

**Affiliations:** 1 Neuro-Psychiatrisches Zentrum Riem, Munich, Germany; 2 Psychology, Istanbul Mediniyet University, Istanbul, Türkiye

## Abstract

**Introduction:**

Approximately 2.8 million people of Turkish origin live in Germany, about 1.5 million of whom hold German citizenship (Statistisches Bundesamt 2023). Turkish immigrants show elevated rates of mood, anxiety, and somatoform disorders that may impede social integration (Dingoyan et al. 2017). Interpersonal competence—the ability to manage social interactions and build meaningful relationships—plays a key role in integration but can be constrained by language barriers, low acculturation motivation, and limited communication opportunities (Kim 2016). Among Muslim immigrants, perceived religious discrimination constitutes an additional obstacle to interpersonal functioning (Nadeem et al. 2020). We examined whether depressive symptom severity moderates the relationship between perceived religious discrimination and interpersonal competence among Turkish psychiatric outpatients at NPZ Munich.

**Objectives:**

To test whether depression strengthens the association between perceived religious discrimination and reduced interpersonal competence, and to identify sociodemographic correlates of these constructs.

**Methods:**

Ongoing cross-sectional study of Turkish immigrant psychiatric patients (target **n≥100**, age 18–65) participating in group therapy at NPZ Munich. Measures: depression (Beck Depression Inventory; Turkish version validated by Hisli 1988), interpersonal competence (Interpersonal Competence Questionnaire–Short Form; Şahin & Gizir 2001), and perceived religious discrimination (Tuysuz & Cinici 2021). Data are collected via self-report prior to sessions, anonymized, and processed in compliance with GDPR. Analyses use SPSS v21 and Hayes’ PROCESS Macro v4.2 (Model 1) for moderation.

**Results:**

Preliminary Results (n=68)

Depression did not significantly moderate the relationship between religious discrimination and interpersonal competence. Depression was not significantly associated with either variable (all ps>.05). Perceived religious discrimination correlated negatively with interpersonal competence, r(66)=−0.28, p=.003. Recruitment continues toward ~120 participants to ensure power for interaction testing and covariate adjustment.

**Conclusions:**

Preliminary findings suggest that religious discrimination is directly associated with lower interpersonal competence, but this effect does not vary as a function of depression severity. Addressing discrimination in clinical and community contexts—alongside language support and culture-informed psychosocial skills training—may promote integration among Turkish immigrants. Final analyses will report the interaction term with relevant covariates (e.g., length of stay, language proficiency, citizenship status) and assess robustness across subgroups.

**Disclosure of Interest:**

None Declared

